# Relation of the Physician to the Results of Gonorrhœa

**Published:** 1891-11

**Authors:** R. R. Kime

**Affiliations:** Lecturer on Clinical Diseases of Women, Southern Medical College, Atlanta, Geo2gia


### RELATION OF THE PHYSICIAN TO THE RESULTS OF GONORRHŒA.

BY R. R. KIME, M. D.
Lecturer on Clinical Diseases of Women, Southern Medical College, Atlanta, Geo2gia.

Not long since gonorrhœa in the female was considered a very trivial matter as compared with it in the male. Now the opinion is being reversed and justly so, as we hope to demonstrate later on.

So long as women of attractive physique and doubtful virtue exist and man is swayed more by passions and lusts of the flesh than by moral influences or the approving consciousness of a virtuous life, just so long will we have the curse of their sins to deal with. If only the guilty had to suffer it would matter but little, but it is here the innocent has to suffer so often for the sins of the guilty. It is impossible to reach that standard of morals in which all men would be virtuous and women have no occasion to be otherwise, yet it is possible for the medical profession to so enlighten such men as to deter them from indulgence from a fear of disease and suffering not limited to themselves only, if not from a sense of moral obligation.

While some place a high estimate on the usefulness, duty and destiny of mankind and are susceptible of being so influenced by spiritual, moral and social influences as to induce them to lead pure lives, others require visions of danger, disease and suffering to prevent indulgence in what should be sacred to the hymenial altar.

It is here the voice of the physician should be heard in no uncertain tone proclaiming the evil results as they are recognized under our present light and knowledge of this disease.

If the laity, especially the men, fully understood the evil results following gonorrhœa they would be more cautious, often refraining from promiscuous indulgence from fear if not from a moral sense.

The physician is often to blame for not instructing his patient of the evil results likely to follow from an incomplete cure of gonorrhoea in the male. Many a physician should be held responsible for the advice given the husband by which his pure, noble, innocent wife is made to suffer the penalty of his broken vow.

Or for the advice given the young lover about to take unto himself the coveted prize, a noble, innocent young woman, having led a pure chaste life,of whom he could truthfully say:

Her virtues, graced with eternal gifts
Doth breed love's settled passions in my heart.—[Shakespeare.

In return for which he tenderly and lovingly conveys to her (by advice of his physician) the germs of disease, the results of a broken virtue, a thing he would abhor in her,and his only excuse is:

"Ah Vice! How soft are thy voluptuous ways!
While boyish blood is mantling, who can 'scape
The fascination of thy magic gaze ?"—[Byron.

It is here that we have fully demonstrated "where ignorance is bliss 'tis folly to be wise." Far better is it that the "greatest sufferer, not the greatest sinner" should remain ignorant of the cause of her condition, and receive love's embrace, than through wisdom scorn the embrace and turn love into hate and a life made happy through ignorance into a life made miserable through wisdom.

Yet he who sins should not be ignorant, and here the duty of the physician is made plain, and ignorance is not bliss,but a crime. He who deigns to advise should first know, and where knowledge fails give safety the advantage of the doubt.

It is no palliation for a physician to say I do not believe in the germ theory of gonorrhoea, the latent theory of Noegareth or in its transmicability to the female with such serious consequence. His disbelief does not controvert clinical facts, and he should be held none the less responsible for the advice he gives. The responsibility of the physician leads us to the consideration of the following questions:

1st. When is gonorrhoea completely cured in the male ?

2nd. How long should the male refrain from sexual intercourse after an attack of gonorrhoea ?

3rd. What are the results of gonorrhoeal infection in the female ?

In considering the first question the etiology of gonorrhoea is an important factor. If we grant it to be a specific contagious disease due to a micro-organism, then its origin, re-production, habits, developments, effects and decay must be considered in order to arrive at a definite conclusion. The details of which we leave for the bacteriologist to demonstrate, and turn our attention to the clinical history, which demonstrates that where the gonococci of Neiser is found in the urethral discharge gonorrhoea is present, that ordinarily acute gonorrhoea in the male runs its course in about three weeks and then subsides or passes into a sub-acute or chronic form with consequent complications and sequelae, that a great many cases are apparently cured, but weeks or months afterward,by some local irritation such as sexual excess, a debauch or passage of a sound, re-kindles the attack.

We are forced to admit that the gonococci must have found lodgment in the urethra, and has continued to re-produce itself, else its germ was retained awaiting the proper stimulus to complete its development and renew the attack, and during the interim, we are also forced to admit, that the sexual intercourse would be likely to communicate the disease.

As to how long this danger of transmission may exist depends on the condition of the urethra as to local points of inflammation, ulceration, deposit of hyperplastic connective tissue exudate, and developing true stricture, forming suitable environment and nidus for the protection and development of the gonococci; which is the duty of the physician to diagnose and remove before declaring his patient cured and permitting sexual intercourse. No physician is warranted in advising marriage after gonorrhoea until all discharge has ceased, a sound introduced several times and the gonococci demonstrated to be absent. This leads us to consider the effect of gonorrhoea in the female, which depends on the activity of the virus introduced, the part affected and its susceptibility to the influence of the poison.

In the male we have one avenue of entrance, different grades of inflammation and various complications, while in the female we have double the avenues of entrance, double the grades of inflammation and double the number of complications, so we should be twice as cautious in prevention and doubly energetic in treatment. It is here "an ounce of prevention is worth a pound of cure."

Gonorrhoeal virus developed in the acute, active, progressive stage of inflammation is evidently more potent for evil than virus developed in the retrogressive declining stage. The virus developed in a light attack of gonorrhoea in the male and during the declining or apparently quiescent stage conveyed to the female whose genetilia are in a normal, healthy condition would be most likely to develop a light grade of urethritis, vulvitis, vaginitis, cervicitis, endo-metritis and salpingitis, very slow in accomplishing its results, and might even run its course for lack of vitality before it reaches the uterine cavity, while upon the other hand, if the virus be developed in the acute, active, invasive stage we would naturally expect as equally active grade of inflammation in the female, running its course more rapidly and more serious in its results.

It is equally as reasonable to suppose we can have these various grades of gonorrhoea in the female often developing salpingitis, ovaritis, pyosalpnix and pelvic peritonitis resulting in death, as that we can have scarlatina attacks so light as to be scarcely diagnosable to those severe forms with dangers, complications, sequelae and death, or that we can have those manifestations of malaria, the result of the Bacillus Malariae evidencedby a light chill and slight fever, to those severe and malignant forms resulting in death.

When once the female genetillia are infected it is very difficult to tell where its ravages will cease, especially if virus is of active form and re-inforced by a mixed infection and finds a soil congenial to its development.

Here the gonococci (if any kin to man) certainly revels in pure delight, re-producing its kind and with the newly-formed hosts proceeds to invade new territory, having conquered the vaginal canal, overcomes the obstacle, enters the cervical canal, (from which some claim he is never routed,) firmly intrenches himself there in and behind the walls of arbor vita, from which he overpowers the internal sentinel, enters the uterine cavity and slowly but surely scales the walls of the chamber of creation until its upmost corners are reached, there by its inherent power and myriad hosts forces its way into the celestial hallway where none but the germs of a higher order of creation should tread.

When once he enters the sacred precincts of this hallway he is safe from the irrigator, curette and antigermicide, and without fear proceeds at once with his work of destruction and ruin, soon destroying the grandeur of the structure and forever annihilating its normal function. Not content with this he fills the hallway full to overflow with an obnoxious fluid which occasionally finds unawares the inner door ajar and escapes through the fringed curtain to a cavity not congenial to its development, and is soon surrounded by inflammatory deposits, or dissolution of the unfortunate individual takes place.

This in brief is the course of the gonococci in the female if not interfered with by nature or the physician.

It is plainly the duty of the physician to check the gonorrhoea while confined to vaginal canal, failing in this reach it in cervix or uterine cavity, for when it reaches the fallopian tubes it is beyond the reach of the physician, and can only be removed by the knife successfully except in rare cases.

While an attack of acute gonorrhoea in the female affecting the urethra, vulva, or vagina may be easily diagnosed, it is a more difficult matter to recognize these lighter attacks, especially when they assume a more chronic form and invade more especially the cervical canal, uterine cavity and fallopian tubes, or a partially controlled acute attack left to invade these structures.

It is as reasonable, even more so, to suppose that the gonococci may intrench itself in the cervical canal and retain its vitality and power of re-production as long in the male urethra and with as little or less manifestations of its presence so long as confined to cervix. Quite different the effect when the gonococci enters uterine cavity and fallopian tubes, with the clinical history of which you are all familiar. It is the duty of every gynecologist to inquire well into the history of his cases and have pus tubes and abscess walls as well as the contents examined microscopically, so as to arrive at a more definite conclusion as to what per cent. are due to gonorrhoeal infection. It is clearly the duty of the accoucher in cities especially, to investigate closely septic infection following abortion, and labor to see what influence gonorrhoeal infection has in the development of the septic condition.

This subject we leave for future consideration, and close our remarks by saying—it is criminal for a physician to advise marriage or sexual intercourse until all discharges from urethra have ceased, a sound has been introduced into urethra to see if it will rekindle the attack, or the gonococci has been demonstrated to be absent by use of the microscope.

